# Phenotypic analysis with *trans*-recombination–based genetic mosaic models

**DOI:** 10.1016/j.jbc.2023.105265

**Published:** 2023-09-19

**Authors:** Yu Zhang, Jianhao Zeng, Bing Xu

**Affiliations:** 1 School of Life Sciences, Nantong University, Nantong, Jiangsu, China; 2 Department of Microbiology, Immunology, and Cancer Biology, University of Virginia Health System, Charlottesville, Virginia, USA

**Keywords:** MARCM, MADM, *Drosophila*, mouse, zebrafish, lineage tracing, cell–cell interaction, genomic imprinting, cancer

## Abstract

Mosaicism refers to the presence of genetically distinct cell populations in an individual derived from a single zygote, which occurs during the process of development, aging, and genetic diseases. To date, a variety of genetically engineered mosaic analysis models have been established and widely used in studying gene function at exceptional cellular and spatiotemporal resolution, leading to many ground-breaking discoveries. Mosaic analysis with a repressible cellular marker and mosaic analysis with double markers are genetic mosaic analysis models based on *trans*-recombination. These models can generate sibling cells of distinct genotypes in the same animal and simultaneously label them with different colors. As a result, they offer a powerful approach for lineage tracing and studying the behavior of individual mutant cells in a wildtype environment, which is particularly useful for determining whether gene function is cell autonomous or nonautonomous. Here, we present a comprehensive review on the establishment and applications of mosaic analysis with a repressible cellular marker and mosaic analysis with double marker systems. Leveraging the capabilities of these mosaic models for phenotypic analysis will facilitate new discoveries on the cellular and molecular mechanisms of development and disease.

Genetic mosaicism, characterized by the coexistence of cells with distinct genotypes within an individual, contributes to cellular heterogeneity and plays a fundamental role in development, cancers, and neurological diseases (1, 2, 3). Genetic mosaic models enable studies of gene function in specific cell types at a desirable time, without disturbing other cell types and overall development. Recombinase-mediated conditional knockout (CKO) strategy, including Cre/loxP, FLP/FRT, and Dre/rox recombination systems (4, 5, 6, 7, 8), is the most widely used approach to generate a genetic mosaic model. Through cell type–specific promoter-controlled Cre or Cre-ER^T^ (fusion of Cre with the mutated ligand-binding domain of the human estrogen receptor, which is activated by tamoxifen), the gene of interest, which is floxed by loxP sites, can be deleted and studied with high spatial and temporal resolution (8, 9, 10, 11). The CKO method has been widely used to study genes’ function in both normal development and various diseases (12, 13). For example, CKO of tumor suppressor genes is used to model many human cancers (14). These tumor models have substantially contributed to our understanding of the molecular and cellular mechanisms underlying tumorigenesis.

However, the CKO models also present considerable limitations in phenotypic analysis. First, the identification of the mutant cells by the reporter is unfaithful. Various methods have been developed to achieve mutant cell labeling in CKO models, including independent Cre reporter (15, 16), insertion of marker genes downstream of the floxed allele (17), or Cre-induced inversion-mediated gene mutation and reporter expression (18). Due to the stochastic nature of two independent recombination events (*i.e.*, gene knockout & turn-on of the reporter), these methods cannot guarantee 100% coupling of gene knockout and labeling (19). Moreover, the expression of the reporters relies on the endogenous promoter, which may be off or too weak at the time of analysis. Second, CKO models mostly achieve tissue-level rather than single-cell-level manipulation of genes of interest, lacking the resolution for analyzing clonal behavior and distinguishing cell-autonomous from cell-nonautonomous gene functions. Third, in CKO models, there is a lack of visible internal control cells for phenotypical comparison. Additional wildtype animals are needed for dissection of mutation-caused phenotypes in CKO models, demanding a large number of animals to reach statistical significance. Thus, novel genetic mosaic analysis models that overcome these limitations are needed for more precise phenotypic analysis.

In *Drosophila*, previous studies utilized the Flp-mediated mitotic interchromosomal recombination at homologous chromosomal loci containing the FRT sites to generate genetically mosaic models, which create sparse mutant cells in the wildtype background to facilitate the mosaic analysis and genetic screening (20, 21). The cell type–specific promoter-controlled FLP can achieve cell-specific labeling in a temporally controlled manner. This mitotic recombination-based mosaic analysis offers several advantages over the aforementioned CKO animal models, including single-cell labeling, 100% coupling between color and genotype, and no requirement for floxed alleles. However, the original *Drosophila* mosaic system is a negative labeling system, in which the mutant cells lose the marker gene and become unlabeled, hampering the tracing of the mutant cells' behavior.

To address these challenges, further improved genetically mosaic analysis models were developed, including mosaic analysis with a repressible cell marker (MARCM) (22) and related twin-spot MARCM (23) in *Drosophila* and mosaic analysis with double markers (MADM) in mouse and zebrafish (24, 25, 26). These models utilize the same recombinase-mediated *trans*-recombination principle but introduce a positive labeling of mutant cells. Therefore, these advanced mosaic analysis models additionally enable precise tracing of the mutant cells of interest with high spatiotemporal resolution. This capability has substantially facilitated the studies of developmental process, genetic imprinting, cell–cell interaction, and disease progression and has led to numerous ground-breaking discoveries (27, 28, 29, 30). In this review, we will revisit the principles and establishment of *Drosophila* MARCM and mouse MADM systems and highlight the applications of these mosaic analysis models. Finally, we will summarize the advantages and limitations of these mosaic models and their future directions.

## Principles of *trans*-recombination-based genetic mosaic models

### Mosaic analysis with a repressible cell marker

**Figure 1:**
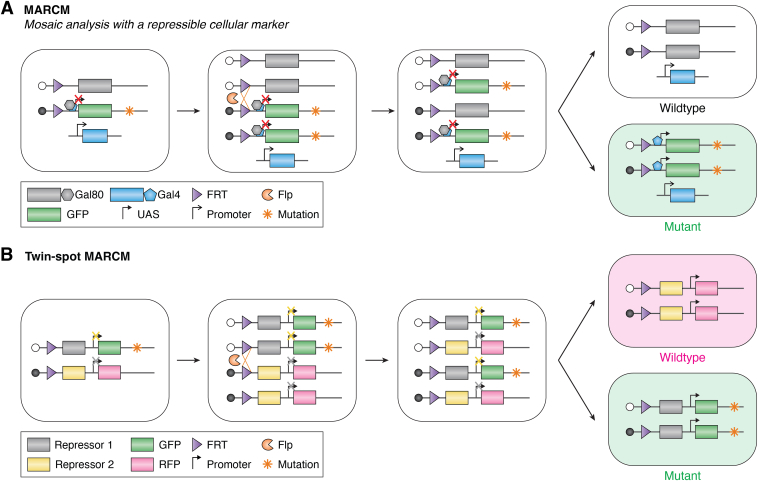
**The principles of *Drosophila* MARCM and twin-spot MARCM.***A*, the Gal80, which can repress the expression of marker gene, is placed at the *trans* site of marker gene. The mutated gene is linked to the marker gene. After Flp-mediated mitotic interchromosomal recombination, the daughter cell with gene mutation will lose the Gal80 expression and is labeled with marker gene, while another sibling wildtype cell with Gal80 is still unlabeled. *B*, two incompatible repressible binary expression systems in the mother cell repress the expression of both marker genes independently. The Flp-mediated interchromosomal recombination leads to the separation of these two repressible systems into different cells, which derepresses the expression of marker genes and label the two daughter cells with different colors. MARCM, mosaic analysis with a repressible cell marker.

To positively label the mutant cells, the repressible binary system was introduced into the traditional mosaic genetic system to establish the MARCM. The mutated gene of interest is in *trans* with the repressor. After the Flp/FRT-mediated interchromosomal mitotic recombination and segregation, one of the progeny with the gene mutation will lose the repressor, leading to positive labeling, while another wildtype progeny still contains the repressor and is unlabeled (22) (Fig. 1*A*). The original repressible binary system is Gal80, which can repress the marker gene expression driven by Gal4. Only the cells with Gal4 and without Gal80 can be labeled (Fig. 1*A*). To achieve cell-type–specific labeling, tissue-specific promoters can be introduced to control the Gal4 expression. For temporal control of MARCM labeling, a heat-shock inducible Flp system can be leveraged. In addition to Gal4-Gal80, other repressible binary systems, like the Q system (31) and RNAi system (23), have been introduced to MARCM to broaden its usage under different experimental scenarios. Nevertheless, all these MARCM systems label the mutant daughter cells only, the unlabeled sibling wildtype cells fail to serve as the internal control for lineage tracing and genetic studies. To overcome this weakness, twin-spot MARCM was developed by using two incompatible repressible binary expression systems and dual reporter genes (GFP and RFP) to label the two daughter cells with different colors (23, 31) (Fig. 1*B*). In the mother cell of a twin-spot MARCM system, the two repressors placed at the *trans* sites of homologous chromosomes independently repress the expression of both reporter genes. The mitotic interchromosomal recombination and segregation causes the loss of the corresponding repressors in the daughter cells, leading to the expression of reporter genes (Fig. 1*B*). To further extend the usage of the MARCM system, Gal4/UAS or LexA/lexAop binary expression systems can combine with the Gal80-based MARCM to overexpress the gene in the mutant cell to do the rescue or other genetic manipulation to comprehensively study the genes’ function (32, 33).

### Mosaic analysis with double markers

**Figure 2:**
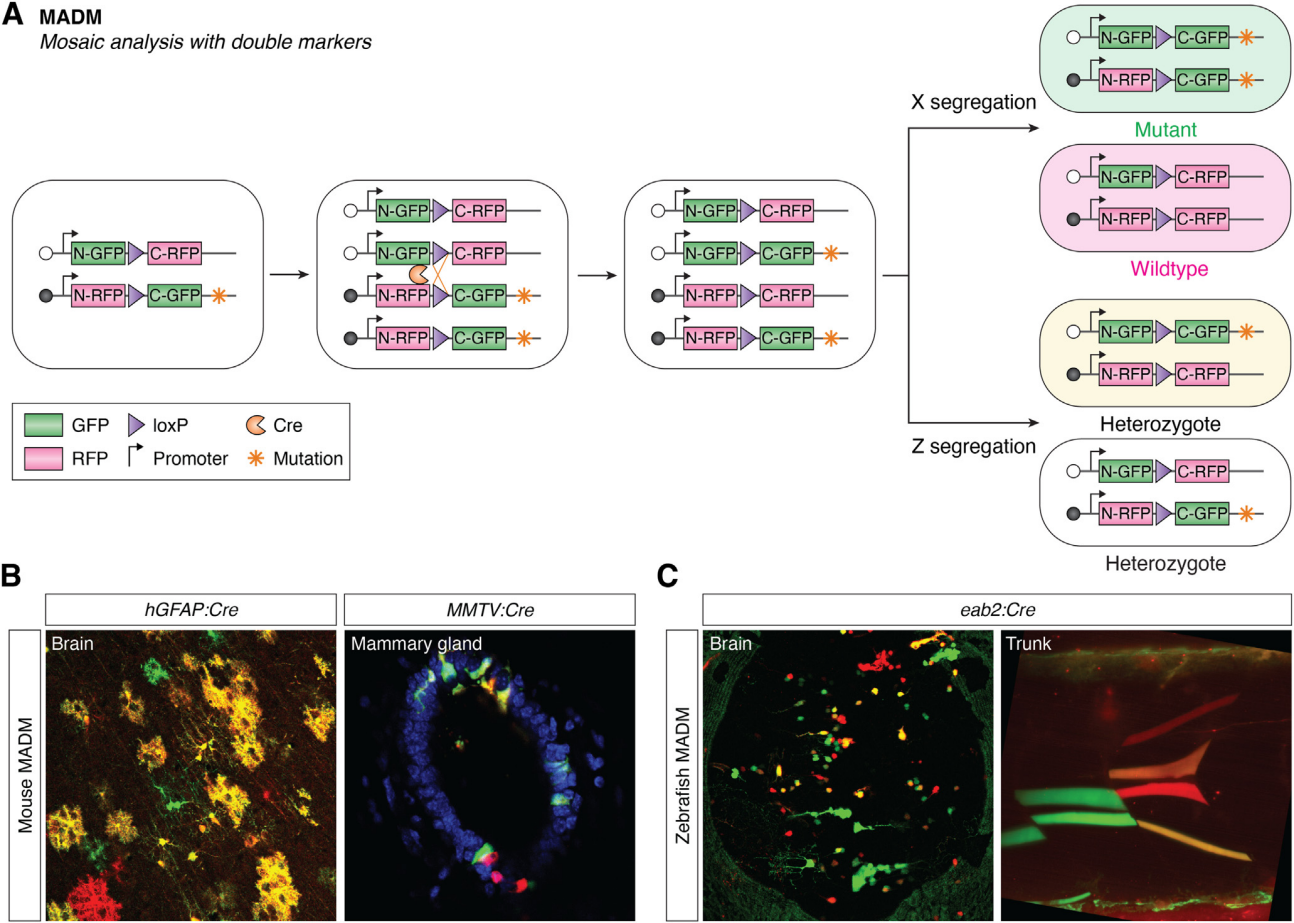
**The principle and labeling of MADM.***A*, the reciprocal chimeric reporter genes are inserted into the identical loci of homologous chromosomes. The mother cell is colorless since there is no functional proteins expressed. After the Cre-mediated interchromosomal recombination and segregation, the daughter cells will be labeled with different colors as the expression of functions proteins. The linkage of gene mutation with the distal part of chimeric reporters leads to the labeling of homozygous mutant cell with different color from the sibling wildtype cell. *B*, the single-cell labeling in the brain (*hGFAP:Cre*) and mammary gland (*MMTV:Cre*) by mouse MADM system. *C*, the single-cell labeling in the brain and trunk (*eab2:Cre*) by zebrafish MADM. MADM, mosaic analysis with double marker. *hGFAP:Cre*, human glial fibrillary acidic protein-controlled Cre; *MMTV:Cre*, mouse mammary tumor virus-controlled Cre.

Different from the MARCM system, MADM utilizes the reciprocal chimeric reporters, instead of the repressible binary system to achieve cell labeling. To establish mouse MADM, two cassettes containing reciprocal chimeric marker genes driven by a ubiquitously expressed promoter are separately inserted into identical loci on homologous chromosomes (24). The chimeric marker genes contain partial sequences of GFP and RFP, which are separated by artificial introns with LoxP sites. Before recombination, all cells remain colorless due to the absence of functional reporters. After Cre-mediated mitotic recombination, the functional reporters are reconstituted (Fig. 2*A*). Upon different segregation patterns of the chromosomes, the daughter cells will show different colors. When the recombinant chromosomes are segregated into different daughter cells (X segregation), a GFP-expressing (green) cell and its RFP-expressing (red) sibling are generated. Alternatively, when the recombinant chromosomes are segregated into the same daughter cell (Z segregation), a GFP and RFP dual-expressing (yellow) cell and a colorless sibling are generated. When a mutant allele of a gene of interest is syntenic with one MADM cassette, *i.e.*, the N-RFP-C-GFP, the green cell generated will be homozygous mutant, the sibling red cells will be wildtype, and the yellow cells will be heterozygous (Fig. 2*A*). Specific promoter-controlled *Cre* can be used to label specific cell type with MADM. For example, *hGFAP:Cre* (*human glial fibrillary acidic protein-*controlled *Cre*) mediates the labeling of brain cells; *MMTV:Cre* (*mouse mammary tumor virus*-controlled *Cre*) mediates the cell labeling of mammary gland (Fig. 2*B*). In the MADM system, the gene manipulation and reporter expression are tightly linked in a single recombination event, circumventing the issue of unfaithful coupling of reporter and genotype (two independent recombination events) in CKO models. Initially, MADM was established in the genomic Rosa26 locus on chromosome 6 (24), limiting the usage of MADM in studying genes on chromosome 6 only. Recently, Simon Hippenmeyer’s group generated a library of mice with knocked-in MADM cassettes to all mouse chromosomes (34). With this genome-wide library of MADM resources, >96% of genes can be subjected to single-cell genetic mosaic analysis, which will enable studies of gene function at single-cell resolution across a broad spectrum of scenarios. Furthermore, similar to the MARCM, when combined with the binary expression system, MADM can be used for gain-of-function studies, which will enable more versatile usage of MADM in mouse genetic studies (35).

MADM-based models have also been established in organisms other than mice, including *Drosophila* and zebrafish (25, 36). Zebrafish as a model system holds several unique advantages, including high fecundity, external and fast development of embryos, and embryonic transparency, enabling *in vivo* real-time analysis and drug screening. Although different methods of genetic manipulation have been developed for zebrafish models, the mosaic analysis of gene function remains challenging. The MADM system was recently introduced into zebrafish (zMADM) (25), empowering real-time mosaic analysis. The ubiquitous expressed promoter *eab2*-controlled Cre can be used to label different cell types at the single-cell level, like brain and muscle cells (Fig. 2*C*). As an example, zMADM was used for *in vivo*, real-time lineage tracing of the neuronal column development at the single-cell resolution. Mutations in genes like *neurofibromin 1* (*nf1*, a tumor suppressor gene known for negatively regulating RAS signaling through converting active GTP-bound RAS into inactive GDP-bound RAS (37, 38)) were also successfully introduced into the zMADM system, demonstrating its ability for single-cell gene knockout and phenotypic analysis. We envision zMADM will be a potent tool to study how gene mutation impacts cellular behavior at real-time *in vivo*, which should reveal intricate details of the developmental process and disease mechanisms.

## Applications of *trans*-recombination-based genetic mosaic models

**Figure 3:**
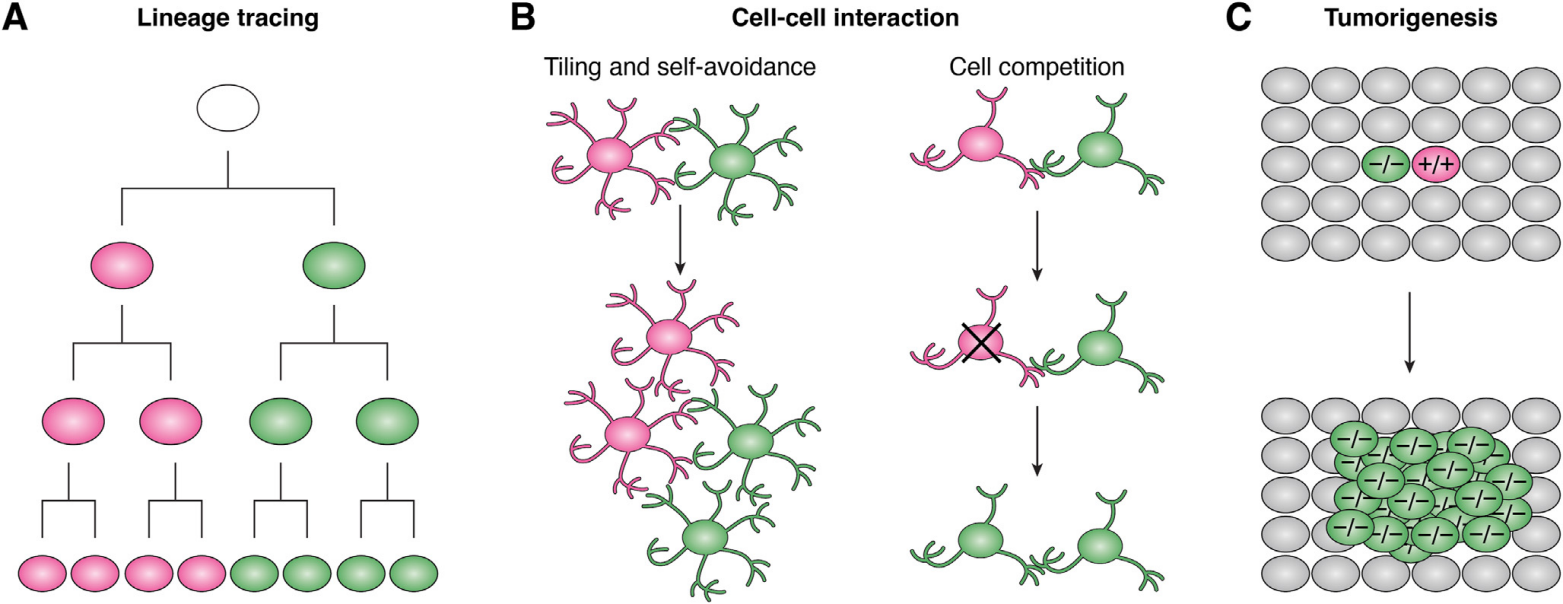
**The applications of MARCM and MADM.***A*, the labeling of sibling cells with different colors is suitable for the lineage tracing. *B*, through analysis the interaction of these two daughter cells, MARCM and MADM can be used for the studies of cell–cell interaction. *C*, the single-cell level labeling of mutant cells is similar to the origin of different cancers, indicating MARCM and MADM can be used to trace the cancer progression. MADM, mosaic analysis with double marker; MARCM, mosaic analysis with a repressible cell marker.

Because the MARCM- and MADM-related models can label the sibling cells with different markers at the single-cell resolution and couple the gene mutation and labeling, they are excellent models for studying lineage development, neural circuit formation, cell–cell interaction, disease progression, and gene function during these biological processes (Fig. 3). Here, we highlight the applications of MARCM- and MADM-related models in different biological processes.

## Application of *Drosophila* MARCM and related models

### Lineage tracing and neural circuit formation

The permanent labeling of sibling cells with different colors makes MARCM and related models suitable for precise dual-lineage tracing in studies of development (Fig. 3*A*). The stereotypic development of the *Drosophila* olfactory system offers an excellent model for the studies of neuronal development and circuit formation (39, 40, 41).

Time-lapse imaging of MARCM-labeled single olfactory receptor neuron (ORN) revealed (1) the early arriving ORN axons target the posterior glomeruli, while the late-arriving ORN axons target the anterior glomeruli and (2) ORN axons are required for the refinement of projection neuron (PN) dendrites but not required for the maintenance of PN dendrites (42). For the development of PNs, MARCM and twin-spot MARCM-based comprehensive analysis revealed that PNs are derived from three separate neuroblast lineages (anterodorsal, lateral, and ventral) and are generated sequentially in a stereotyped order (23, 43, 44, 45). In every lineage, the asymmetric division of a neuroblast generates a new neuroblast and a ganglion mother cell, which finally generates two mature neurons (22). Further characterization demonstrated only one postmitotic neuron survives and develops into a PN in the anterodorsal and ventral lineages, while both postmitotic neurons survive and develop into a PN and a local interneuron in the lateral lineage (46, 47). The *Drosophila* mushroom body contains different intrinsic neurons, known as Kenyon cells, which are classified as γ, α′/β′, or α/β neurons. The MARCM-based single clonal analysis demonstrated that these neurons are generated sequentially, with γ neurons first, followed by α′/β′ neurons, and the α/β neurons are generated last (48). All of these results demonstrate the MARCM and related models are great mosaic models for studying the developmental process with high spatial and temporal resolution.

### Cell–cell interaction

The sparse labeling of twin-spots of wildtype and mutant cells with distinct colors and close physical proximity is a powerful tool to study the detailed mechanisms of cell–cell interaction, including self-avoidance, tiling, and cell competition, etc. (Fig. 3*B*). Tiling and self-avoidance are critical for the dendrites of neurons to cover the sensory field completely without redundancy. Tiling refers to the nonoverlapping coverage of dendrites between the same classes of neurons. Self-avoidance refers to the tendency of neurites from the same neuron to avoid crossing each other, thereby promoting branch segregation and the establishment of a uniform sensory field (49). In the developing *Drosophila* epidermis, the dendrites of different dendritic arborization neurons form overlapping pattern, whereas sister dendrites do not, providing an excellent model to study the mechanisms of dendritic tiling and self-avoidance (50, 51). Using the MARCM system, the dendritic arborization neurons are classified into four morphological classes, of which neurons from two classes cover the body wall with a nonoverlapping, tiling pattern, while the dendrites of neurons in different classes overlap each other extensively (50). The sister dendrites from the same neurons display self-avoidance. The tiling phenomenon is also observed in the *Drosophila* visual system (52). Further analysis with MARCM combined with gene mutation illustrated the molecular mechanisms for tiling and self-avoidance. For example, the dendrites of the *starry night* (*stan*) mutant project beyond their territories (50). The mutation of *Furry* (*Fry*) and *Tricornered* (*Trc*) kinase causes the massive terminal branching of dendrites and the defects of self-avoidance, indicating that *Trc* and *Fry* are essential for tiling and dendrite branching control (53). Furthermore, MARCM-based studies identified the genes of Down syndrome cell adhesion molecule (Dscam) family play critical roles in the self-avoidance and tiling through homophilic interactions. For example, the sister dendrites from the same neurons, expressing the same isoforms of Dscam, exhibit self-avoidance. *Dscam2* mediates the axonal tiling in the *Drosophila* visual system. Loss or overexpression of Dscam disrupts the uniform pattern of dendritic branches (51, 52, 54, 55).

MARCM-based single-cell gene mutation is also used to study the mechanisms of cell competition, which plays important roles in tissue homeostasis, development, aging, and cancer progression (56). Cell competition was first found that the *Minute*^*+/−*^ (encoding ribosomal proteins) cells were viable in a homogenous context but were outcompeted when introduced into the wildtype *Drosophila* (57). Using the MARCM system, Li *et al.* (58) found that engulfment is required for the wildtype cells to outcompete and eliminate the *Minute*^*+/−*^ cells. Furthermore, multiple engulfment genes, including *draper, wasp, the phosphatidylserine receptor, mbc/dock180*, and *rac1*, are involved in this cell competition process. Chen *et al.* (59) reported cell competition between tumor suppressor gene (*Scrib*) mutant and wildtype cells prevents hyperproliferation of the *Scrib* mutant cells, leading to the suppression of tumor formation. Nagata *et al.* (60) showed autophagy drives the elimination of the loser cells *via* NFκB-mediated Hid and Junk expression during cell competition.

## Applications of mouse MADM

### Developmental studies

The mouse MADM system has been widely used for studying the development of various organs, such as the nervous system, kidney, cardiac system, etc. (29, 30, 61, 62). Analysis of the excitatory neurogenesis in the mouse neocortex with MADM revealed every individual radial glial progenitor (RGP) generates 8 to 9 neurons, distributing in both deep and superficial layers. Upon mutation of *orthodenticle homolog 1* (a homeodomain transcription factor transiently expressed in RGPs), the neuronal number and unit size both decreased by decreasing the neurogenic capacity (63). Another study of the neuronal organization in the neocortex with MADM revealed a diverse combination of clustered protocadherins expressed in individual excitatory neurons regulates the fine organization of the neocortex. The expression of functional clustered protocadherin affects the clustering of clonally related excitatory neurons originating from the same neural progenitor and synaptic connectivity (64). MADM has also been used to study hippocampus development, Shi *et al.* showed that clonally related excitatory neurons are organized into discrete horizontal clusters in the stratum pyramidale. The sister excitatory neurons in the CA1 region exhibit synchronous synaptic activity *via* receiving the common synaptic input from fast-spiking interneurons, instead of forming electrical or chemical synapses with each other (65). Studies of the development and organization of thalamic nuclei with MADM showed individual RGPs generate a cohort of neuronal progeny, showing a functionality-related spatial configuration and nuclear occupation (66). For example, the anterior cluster shows more tangential display and mainly contributes to the nuclei related to cognitive functions, while the medial ventral posterior cluster shows prominent radial arrays and mostly contributes to nuclei with sensory- or motor-related functions. Furthermore, the first and higher-order sensory and motor nuclei across different modalities are largely segregated clonally. This mechanistic study demonstrated sonic hedgehog signaling influences the spatial organization of thalamic neurons (66).

MADM can also be employed to dissect whether a gene functions in a cell-autonomous or non-cell-autonomous manner, owing to its capacity for direct phenotypical comparison (*i.e.*, altered migration, clonal expansion, or gene expression) of mutant cells and their sibling wildtype cells. Attributed to this strength, several important discoveries regarding the role of genes in dendritic development, neurogenesis, gliogenesis, neuronal migration, *etc*. have been made: (1) *N-methyl-D-aspartate-type glutamate receptors 2B* is required for the dendritic patterning, which demonstrates activity-dependent dendrite patterning is regulated differently from general dendrite growth and branching (67); (2) *Lissencephaly-1* (*Lis1*) and *nuclear distribution gene E-like homolog 1* (*Ndel1*) regulate neuronal migration differently. For example, *Lis1* regulates neuronal migration in a dose-dependent manner, while Ndel1 regulates neuronal migration into the final target laminae. This study corrects the previous notion that the LIS1/NDEL1 complex regulates all steps of neuronal migration cell autonomously and also provides novel insights about the cell-autonomous and nonautonomous functions of LIS1 and NDEL1 in regulating neuronal migration (68); (3) *lethal giant larvae homolog 1* regulates the embryonic neurogenesis and postnatal gliogenesis, which reveals distinct sequential non-cell-autonomous and intrinsic cell-autonomous functions of *lethal giant larvae homolog 1* in controlling cortical neuron and glia genesis (69); and (4) *specificity protein 2* regulates late neurogenic but not early expansive divisions of neural stem cells, which reveals mechanistic differences between the early expansive and later neurogenic periods of cortical development (70).

### Genomic imprinting

In diploid genomes, a subset of genes is expressed by only one parental (maternal or paternal) allele, while another allele is preferentially silenced, a concept termed genomic imprinting. Genomic imprinting plays important roles in embryonic development and relates to many diseases (71, 72). Because MADM is based on the *trans*-recombination, the daughter cells derived from the G2-X segregation contain only maternal or paternal alleles, facilitating the functional studies of genomic imprinting in development and diseases. Hippenmeyer *et al.* established uniparental disomies (UPDs) *via* MADM to study the effects of genomic imprinting with single-cell resolution. They found chromosome 7 (Chr. 7) UPD caused significant paternal dominance effects in the liver and lung, including the expansion of liver hepatocytes and lung epithelia, and the *insulin-like growth factor 2* in Chr. 7 mediates most of the paternal dominance effects (73). The study of genomic imprinting in the cerebral cortex development with MADM UPDs and single-cell RNA sequencing demonstrated the cell type–specific expression of imprinted genes. With the Chr. 7 UPD, compared with maternal UPD, the paternal UPD increases the proliferation of astrocytes (74). The study of imprinted *cyclin-dependent kinase inhibitor 1C* (*Cdkn1c*) locus, exhibiting maternal expression, showed Cdkn1c promotes cell growth and survival in a cell-autonomous manner, which is different from the previously reported growth-inhibitory non-cell-autonomous role of *Cdkn1c*. This work highlights the importance of distinguishing the cell-autonomous from the non-cell-autonomous functions of genes to the overall phenotype (75).

### Cell–cell interaction

Mouse MADM has also been used to study the mechanisms of cell–cell contact since the sibling cells can be labeled with different colors. Astrocytes in the mouse brain display nonoverlapping territories. MADM-based analysis revealed a greater extent of overlapping territories between HepaCAM-mutant and wildtype astrocytes, in comparison to wildtype and wildtype astrocytes (76). In the developing brain, Sun *et al.* found cell competition between neural progenitor cells is required to maintain cell fitness and optimize brain size. Through MADM-mediated single-cell gene knockout, they found *Axin2*-deficient NPCs became loser cells and underwent *p53*-dependent cell elimination, while *p53*-deficient NPCs behaved as winner cells. This study demonstrates that the *Axin2-p53* axis coordinates to regulate cell competition and optimize brain size (77). Attributed to the differential labeling of sibling cells, the MADM system will serve as a potent tool for investigating cell–cell interactions between sibling cells or between siblings and surrounding cells.

### Cancer

Most cancers originate from a single cell with gene mutations. MADM generates single mutant cells at a low frequency, thereby mimicking the human cancer initiation from sporadic mutant cells (Fig. 3*C*). The permanent labeling of rare mutant cells with GFP in MADM allows spatiotemporal analysis of mutant cell behavior at any point of tumorigenesis at clonal resolution. Moreover, the RFP+ wildtype sibling cells serve as a perfect internal reference, empowering the detection of subtle abnormalities of mutant cells. Thus, MADM is a powerful tool to study cancer initiation and premalignant progression.

Through introducing *Nf1* and *p53* mutations into MADM, Liu *et al.* established the MADM glioma model and found oligodendrocyte progenitor cells (OPCs) are the cell-of-origin of glioma *via* quantifying the green (mutant)/red (wildtype) cell number ratio during gliomagenesis (78). Further analysis with a single gene mutant in MADM demonstrated the distinct roles of *Nf1* and *p53* in the glioma progression, with mutation of *Nf1* leading to the expansion of mutant OPCs, while *p53* mutation is necessary for the malignant transformation of mutant OPCs to form glioma (79). Furthermore, in this glioma model, the authors found that the knockout of *insulin-like growth factor I receptor* only decreased the proliferation of mutant OPCs, but not normal OPCs, which provides a new avenue for glioma prevention (80). Interestingly, combining the MADM glioma model and specific *IGF1* gene knockout in the olfactory system demonstrated the olfactory sensory experience affects gliomagenesis *via* secreting IGF1 (81). Besides gliomagenesis, MADM has also been used to study the evolution of medulloblastoma and showed tumor cells transdifferentiate to astrocytes, which promote the cancer progression *via* increasing the release of IGF1 from the tumor-associated microglia (82). When combined with *breast cancer gene 1* and *p53* mutations, a MADM breast cancer model was established. Studying the premalignant progression of breast cancer with this MADM model revealed several stereotyped premalignant lesions and identified a partial luminal-to-basal transitional state during cancer development (83). A MADM-based model for fallopian tube-derived ovarian cancer was also recently established. With the clonal and temporal resolution of MADM, the authors revealed that only a rare stem/progenitor-like population in the distal fallopian tube presents ovarian cancer-initiating potential. These cells manifest biased differentiation during cancer premalignant progression (84).

## Summary and perspectives

Genetic mosaic models present invaluable tools for studying gene function in lineage development, neural circuit formation, cellular interactions, genomic imprinting, and tumorigenesis in a spatiotemporal-controlled manner. In addition to the aforementioned *trans*-recombination–mediated mosaic analysis models, numerous *cis*-recombination-based models have also been developed, including brainbow, zebrabow, mosaic mutant analysis with spatial and temporal control of recombination, etc. (85, 86, 87, 88, 89, 90). These mosaic analysis models have provided deep insights into gene function in development and various diseases (91, 92). Comparing to *cis*-recombination-mediated mosaic models, MARCM- and MADM-related mosaic models present unique advantages for single-cell phenotypic analysis: (1) the mutant cells can be faithfully traced by the reporter, because the gene manipulation and labeling occur in a single step, guaranteeing a tight coupling of gene mutation and labeling; (2) attributed to the single-cell resolution, these models can distinguish the autonomous from nonautonomous roles of the genes of interest; (3) as the homozygous and wildtype sibling cells are labeled with different colors in the same animal, the labeled wildtype cells serve as perfect internal control; (4) conditional gene knockout can be accomplished by introducing a single null allele of the gene of interest, bypassing the time-consuming step of generating floxed alleles; (5) due to the modular nature of these systems and the availability of genome-wide libraries of mice with the mosaic cassettes knocked into each of the mouse chromosomes, these mosaic models can be used to study the function of most genes throughout the genome. With these advantages, the MARCM- and MADM-related models should present invaluable tools to acquire deep insights into gene function in development and diseases.

However, some limitations still remain: (1) the low labeling efficiency advantageously facilitates single-cell analysis; however, it also poses difficulty in labeling target cells of a small population; (2) the generation of green mutant and red wildtype cells relies on mitotic interchromosomal recombination; therefore, postmitotic cells cannot be genetically manipulated by these systems; (3) gain-of-function gene studies remain challenging with these models. Despite these limitations, the superior advantages of these *trans*-recombination–based genetic mosaic models make them invaluable for precise phenotypic analysis and comparative assessment of mutant *versus* heterozygous and wildtype cells at the single-cell resolution. Importantly, when used together with other genetic models, the full potential of these genetic mosaic models can be further unleashed in studies of the intricate mechanisms underlying development and diseases.

## Conflict of interest

The authors declare that they have no conflicts of interest with the contents of this article.
